# Successful Treatment of Bullous Pemphigoid Lesions by Berberine Stamp Therapy: A Case Report and Literature Review

**DOI:** 10.3389/fmed.2022.938761

**Published:** 2022-07-01

**Authors:** Linyan Cheng, Yi Wang, Hanzhi Lu, Wanjun Guo, Ge Yan, Jianyong Zhu, Dongjie Guo, Fulun Li

**Affiliations:** ^1^Department of Dermatology, Yueyang Hospital of Integrated Traditional Chinese and Western Medicine, Shanghai University of Traditional Chinese Medicine, Shanghai, China; ^2^Clinical Laboratory Medicine Center, Yueyang Hospital of Integrated Traditional Chinese and Western Medicine, Shanghai University of Traditional Chinese Medicine, Shanghai, China

**Keywords:** bullous pemphigoid, autoimmune dermatoses, berberine, stamp therapy, treatment, case report

## Abstract

Bullous pemphigoid (BP) is a life-threatening autoimmune disease of the skin that is mainly characterized by a large range of tension blisters and intense itching of the skin. The 1-year mortality rate of BP was 23.5%. Superinfection caused by skin lesion ulceration is one of the important causes of disease death. Therefore, it is challenging to control infection and improve skin wound healing. Here, we report the case of an elderly woman who presented with BP and involved the oral mucosa. The patient was successfully treated with hormones combined with topical berberine, and 95% of the patients’ lesions healed completely after 1 month. In addition, we inductively analyzed the current treatments for BP to provide a reference for BP clinical treatment.

## Introduction

Bullous pemphigoid (BP), a type of pemphigoid, is a rare, cutaneous autoimmune disease characterized by autoantibodies against hemidesmosome structural proteins at the dermal-epidermal junction. It is mainly characterized by a large range of tension blisters and intense itching (1). Minor oral mucosal lesions may be present in only 10–20% of these patients, and lesions in other mucosal areas are relatively rare (2). The 1-year mortality rate of pemphigoid is 23.5% (3). Superinfection caused by skin lesion ulceration is one of the important causes of disease death (2). Therefore, it is challenging to control infection and improve skin wound healing. Here, we report a case of an elderly woman who presented with BP and oral mucosa involvement. Based on clinical experience, we used steroids combined with topical berberine and achieved highly positive outcomes. We inductively analyzed the current treatment to provide a reference for BP clinical treatment.

## Case Presentation

**FIGURE 1:**
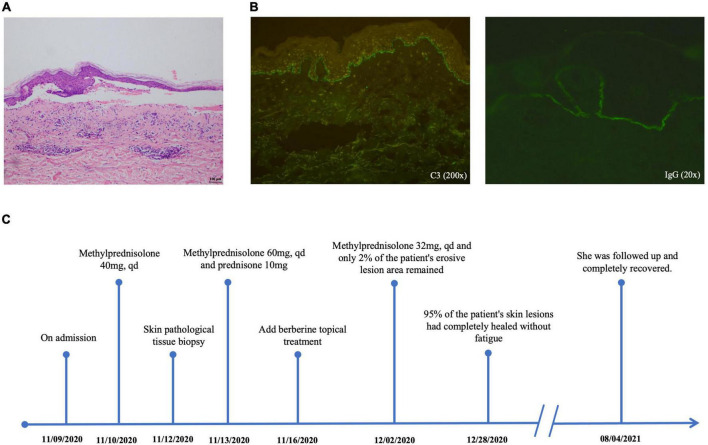
Skin histopathology **(A)** shows that the epidermis is separated from the dermis with blisters in the epidermis (10×). Immunofluorescence staining **(B)** reveals linear C3 and IgG deposits along the basement membrane. Patient timeline **(C)** demonstrates the therapeutic schedule and disease status.

The patient was a 76-year-old woman who had a 5-year history of Sjogren’s syndrome. The first uncomfortable symptom she experienced was a generalized erythematous rash on the skin around the eyes approximately for 10 days, accompanied by increasing intraocular secretions, itching, and blurred vision. A week following the onset of initial symptoms, lesions developed into generalized herpes with itching and partial ulceration on the trunk. Physical examination revealed erythema around the eyes and ulcers on the palate of the mouth, herpes wall laxity, Nikolsky’s sign (±), yellowish herpes discharge, and a thin, partially damaged bright red erosive surface. Laboratory examination revealed the following: desmoglein 1 (Dsg-1), 3.9 U/mL (0.1–200 U/mL); desmoglein 3 (Dsg-3), 100.30 U/mL (0.1–200 U/mL); anti-BP180 antibody, positive; anti-BP230 antibody, positive. Skin histopathology (Figure 1A) showed that the epidermis was separated from the dermis by blisters in the epidermis. Immunofluorescence staining (Figure 1B) revealed linear C3 and IgG deposits along the basement membrane. Combined with its clinical features, we confirmed the diagnosis of BP. After diagnosis, the patient was immediately prescribed the traditional therapy of glucocorticoid steroid methylprednisolone (40 mg per day ivgtt) for symptomatic treatment. Five days post-treatment, new blisters and erosive lesions still formed. Moreover, instead of reducing the methylprednisolone dose, it was also increased to 60 mg per day. According to clinical expertise, traditional Chinese medicine treatments were added to the treatment regimen. Considering the patient’s skin lesions, tongue, and pulse, this condition is classified as damp-heat accumulation skin pemphigus disease. The patient’s treatment timeline is shown in Figure 1C.

**FIGURE 2:**
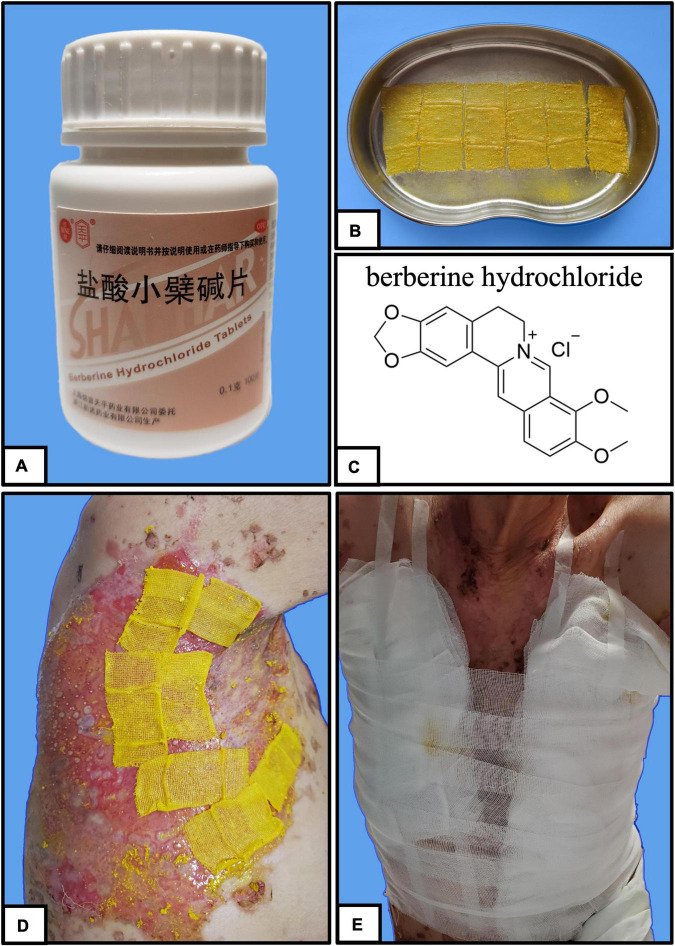
Stamp therapy. **(A–C)** Stamp gauze soaked with sesame oil mixed with berberine. **(D)** Stamp gauze is affixed thoroughly to the broken skin of the patient. **(E)** Bandage is wrapped around the periphery to prevent the gauze from falling.

**FIGURE 3:**
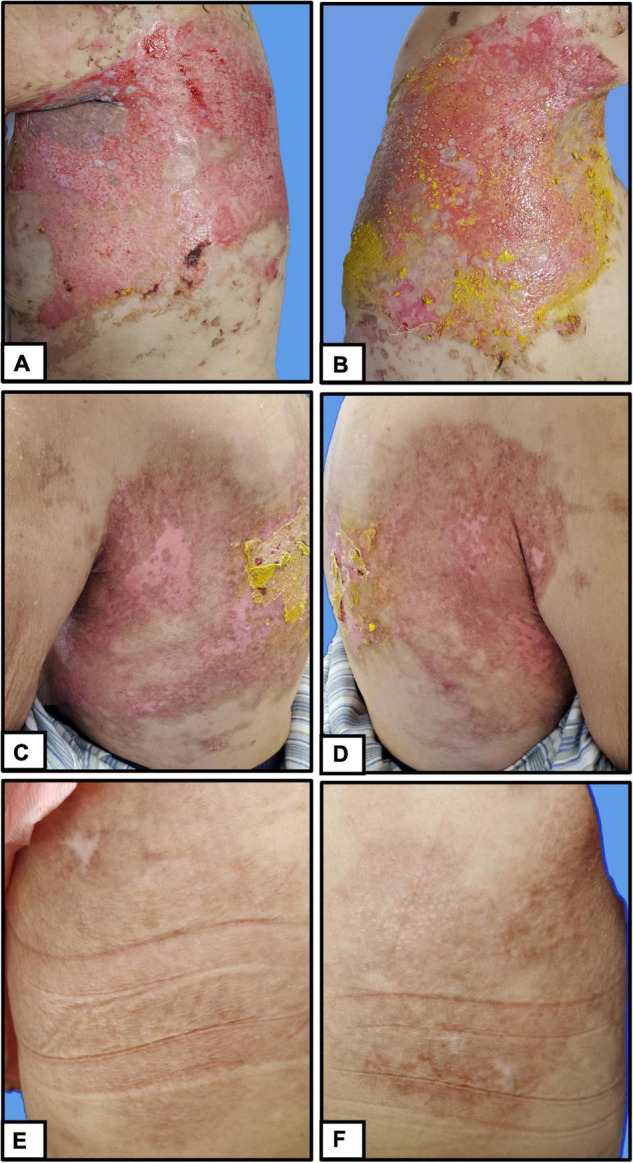
Clinical manifestations of patients with pemphigus. **(A,B)** Before treatment, **(C,D)** 2 weeks after treatment, **(E,F)** during follow-up.

Berberine is primarily derived from *Coptis chinensis* Franch and is typically administered in clinical practice as berberine hydrochloride tablets (Figures 2A,C). It is a common Chinese herbal medicine with the function of heat clearing, dampness drying, and analgesic. Berberine was ground into powder and blended with sesame oil (50 mg/mL) (Figure 2B). Small gauze was immersed in the mixture and was applied one by one to the cutaneous wound in the form of stamps (Figure 2D). Finally, a bandage was wrapped around the outside to prevent the small gauze from falling off (Figure 2E). The outer bandage was opened every 3 days, and most of the small gauze adhered to the eroded skin. Gauze that did not fall off was kept on the skin, and new berberine-soaked gauze was applied onto the skin. Two weeks later, the patient’s periocular and trunk skin lesions were significantly improved, comprising only 2% of the residual surface area of the erosive surface (Figures 3A–D). New epithelial tissue was observed, along with a remarkable pain reduction. Notably, the dose of methylprednisolone was reduced to 32 mg per day. After 1 month of hospitalization, 95% of the patient’s skin lesions had completely healed without fatigue. After hospital discharge, the patient completely recovered from BP and had a great quality of life during follow-up (Figures 3E,F).

## Discussion

In this case, we diagnosed it as BP with the syndrome of damp-heat accumulation skin based on clinical manifestations and skin biopsy. We tried a novel combination of drug and treatment-adjuvant therapy with the external application of berberine, a traditional Chinese medicine extract for clearing away heat, dryness, and dampness. The results showed that this traditional Chinese medicine external method is effective.

Berberine, an alkaloid mainly extracted from the traditional Chinese medicine *C. chinensis*, has hypoglycemic, anti-inflammatory, antioxidant, and broad-spectrum antibacterial properties (4). In clinical trials, berberine has significant improvement effects on hypoglycemia, lipid-lowering, and insulin resistance (5). It also shows significant effects in treating cardiovascular, endocrine, gastrointestinal diseases, and cancer and is mostly used for gastrointestinal diseases such as enteritis and dysentery in clinical practice (1, 5). Berberine has been widely used in research and in the clinic for its anti-inflammatory properties. As early as, Kuo et al. (6) found that berberine can inhibit basal levels and TPA-mediated PGE2 levels and COX-2 expression by inhibiting AP-1 binding, confirming that berberine has a significant potential anti-inflammatory effect *in vivo* (6, 7). Furthermore, berberine may also act on vascular endothelial cells and immune cells via different mechanisms of action (7). It is well-documented that berberine is an effective medicine for the treatment of intestinal inflammatory diseases such as colitis, diarrhea, and dysentery due to its antibacterial activity against *Escherichia coli*, *Helicobacter dysenteriae*, and enterococci (4, 5). At the same time, berberine also has inhibitory effects on other Gram-positive and Gram-negative bacteria, including different strains of *Staphylococcus*, *Actinobacillus pleuropneumoniae*, *Shigella dysenteriae*, and *Streptococcus agalactiae* (5, 7). Moreover, its biological activities against dermatophytes such as *Trichophyton violaceum* and *Microsporum canis* have also been reported and studied (8). Berberine exhibits broad antibacterial effects, and as a natural alkaloid, it has unique advantages in counteracting bacterial resistance. Wojtyczka et al. (9) showed that berberine had significant synergistic effects with linezolid, cefoxitin, erythromycin, and other antibiotics. The authors also suggested that berberine may be an effective tool for the treatment of antibiotic-resistant bacterial infections. Based on the hypoglycemic and anti-inflammatory properties and broad-spectrum antibacterial ability of berberine, Zhang et al. (10) made berberine into the form of a nanohydrogel and explored its effect on wound healing in diabetic rats. The results showed that berberine could indeed effectively promote the repair of diabetic wounds. Samadian et al. (11) also chose berberine as a drug for wound dressing for nursing diabetic ulcers and resulted in a positive effect on promoting wound healing. Aside from antibacterial and anti-inflammatory effects, berberine may also have a certain effect on promoting wound healing.

Bullous pemphigoid is the most common form of pemphigoid, a rare condition affecting the elderly over 70 years of age. Typical clinical features include tension, serous or hemorrhagic bullae with a 1–3 cm diameter on normal or erythematous skin, accompanied by severe itching (12). BP usually occurs in the lateral limbs and lower abdomen. The mucous membrane is involved in 10–20% of patients, and the oral mucosa is more vulnerable than the conjunctiva, nose, pharynx, esophagus, and anus (2). Before forming blisters, there is usually non-bullous pruritus or signs of eczema, papules, and other skin lesions as prodromal symptoms. Such atypical symptoms can easily lead to misdiagnosis. Currently, a detailed medical history plays a vital role, including the specific circumstances of pruritus and the development of lesions, related neurological diseases, malignant tumors, autoimmune diseases and recently used drugs (2, 12). Takegami et al. (13) reported a case of the coexistence of Sjogren’s syndrome and BP in which tension blisters and erosive lesions of the skin were seen and oral mucosa was involved. Although there is no proven link between Sjogren’s syndrome and blistering disease, the patient’s long history of Sjogren’s syndrome and skin lesions around the eyes and mouth, in this case, may induce concern. According to Lucariello et al. (14), there is a certain correlation between BP and malignant tumors based on a meta-analysis. Last, aldosterone antagonists, DPP-4 inhibitors, anticholinergic medications, and dopaminergic medicines have all been linked to an increased risk of developing BP in other related studies (15). Chen et al. (16) meta-analysis concluded that dementia, stroke, heart disease, and diabetes are important factors contributing to the poor prognosis of patients with BP. As a result, special attention should be given to these patients with potential risks. Nevertheless, the diagnosis of BP is based mostly on skin histological biopsy and immunofluorescence confirmation.

At present, the treatment of BP is mainly glucocorticoids supplemented by antibiotics or steroids. According to the German guidelines, the severity of the disease is determined by the extent of skin involvement in BP, with 10% or less being defined as mild, 10–30% as moderate, and more than 30% as severe BP (12). Mild BP should be treated with topical corticosteroids alone, while moderate BP should be treated with other adjuvant therapies in combination with topical corticosteroids, and severe BP should be treated with systemic corticosteroids (12). Clobetine propionate cream (17) is a frequent topical steroid, while oral prednisolone is the most common systemic glucocorticoid therapy (12). In addition to hormones, drugs such as tetracycline and nicotinamide, azathioprine, doxycycline, mycophenolate mofetil, and dapsone are often used as adjuvant or alternative treatments (Table 1; 18–22). Furthermore, according to Engineer et al., intravenous immunoglobulin is an alternative hormone therapy worth investigating because it is effective in the clinic, particularly in resistant BP, with minimal adverse reactions (23). Other treatment options, such as immunoadsorption (24), biological monoclonal antibodies (25), targeted therapy (26), and other approaches, are also worthy of further exploration. In addition to Western medicine treatment, traditional Chinese medicine treatment is currently receiving increasing attention, and its efficacy is considerable. For example, Ge et al. (27) discovered that *Tripterygium wilfordii* Hook F was safe and effective in treating mild to moderate BP, with much fewer adverse effects than hormone treatment.

**TABLE 1:** Summary of clinical studies on adjunct and alternative hormone therapies for bullous pemphigoid (BP).

References	Type of study	Patients	Experimental group	Control group	Result	Conclusion
Kalinska-Bienias et al. (18)	A comparative, retrospective analysis	106	Tetracycline 1.5 g/daily, nicotinamide 1.2 g/daily, 0.05% lesionally administered clobetasol cream (TNC) (*n* = 59)	Prednisone 0.5 mg/kg daily (*n* = 47)	After 4 weeks, 93.2% of patients in the TNC group and 89.1% of patients in the Prednisone group achieved control of their diseases.	TNC is a viable alternative to prednisone treating BP.
Kakuta et al. (19)	A retrospective study	10	Azathioprine 100 mg/day (*n* = 10)	None	70% of patients were successfully treated with Azathioprine monotherapy, with no serious side effects.	Azathioprine monotherapy is an effective therapy for mild to moderate bullous pemphigoid.
Sticherling et al. (20)	An open, multicenter, randomized clinical study	54	Oral methylprednisolone 0.5 mg kg^–1^ and either azathioprine 1.5–2.5 mg kg^–1^ per day (*n* = 27)	Oral methylprednisolone 0.5 mg kg^–1^ and dapsone 1.5 mg kg^–1^ per day (*n* = 27)	Methylprednisolone could be discontinued in eight individuals after a median of 251 days in the azathioprine group (five patients) and 81 days in the dapsone group (three patients).	Dapsone exhibits moderate corticosteroid-sparing effects compared to azathioprine.
Williams et al. (21)	A pragmatic, non-inferiority, randomized controlled trial	253	Oral doxycycline 200 mg per day for 6 weeks (*n* = 132)	Oral prednisolone 0.5 mg/kg per day for 6 weeks (*n* = 121)	At 6 weeks, 74% of doxycycline patients had three or fewer blisters, compared to 91% of prednisolone patients.	Doxycycline is comparable to oral prednisolone for short-term control of bullous pemphigoid and is safer for long-term use.
Beissert et al. (22)	A prospective, multicenter, randomized, non-blinded clinical trial	73	Oral methylprednisolone plus mycophenolate mofetil (mycophenolate mofetil group) (*n* = 35)	Oral methylprednisolone plus azathioprine (azathioprine group) (*n* = 38)	Patients in the azathioprine group achieved complete remission after 23.8 ± 18.9 days compared to 42.0 ± 55.3 days in the mycophenolate mofetil group. Liver function test results elevated induced by azathioprine treatment (*P <* 0.001).	Mycophenolate mofetil and azathioprine have equivalent efficacy in treating bullous pemphigoid. However, the former has lower hepatotoxicity.

In this case, we chose berberine primarily based on traditional Chinese medicine’s basic theory and syndrome differentiation for patients. We considered berberine’s efficacy in clearing away heat and toxic substances, drying dampness, and relieving pain, and the symptomatic treatment of patients with pemphigus of the damp-heat accumulation type. In this case, we used a unique pharmaceutical dosage form called the “stamp therapy” method, from the way gauze adhered to the eroding skin like a stamp (Figure 2D; 28). This method was also a TCM nursing method distilled from clinical practical experience. Studies have shown that this method can effectively protect wounds, promote wound healing, and reduce the suffering herpes zoster patients (28). In this case, the patient’s wound recovery process was smooth, owing to the antibacterial and anti-inflammatory actions of berberine, and its wound healing effects. The usage of stamp therapy for wound care also protected the patient’s large area of blisters and ulcerating, and eroding wounds from serious infection and complications. Despite the lack of a clear understanding of the mechanistic role of berberine in wound healing, these ongoing investigations and clinical results provide a clinical foundation upon which berberine might be further studied and new therapeutic ideas for BP generation.

In conclusion, the advantages of traditional Chinese medicine and external treatment to treat skin diseases are becoming increasingly apparent. Whether as the primary treatment or as a supplementary nursing means, these treatment methods can achieve fantastic outcomes and significantly reduce the occurrence of adverse reactions and complications while reducing pain and improving quality of life.

## Data Availability Statement

The original contributions presented in the study are included in the article/supplementary material, further inquiries can be directed to the corresponding authors.

## Ethics Statement

Written informed consent was obtained from the individual(s) for the publication of any potentially identifiable images or data included in this article.

## Author Contributions

LC and YW participated in writing the manuscript. WG and GY participated in patient management. DG, FL, and JZ contributed to critical revision. HL was responsible for the preparation of the tables and figures. All authors have read and approved the final manuscript.

## Conflict of Interest

The authors declare that the research was conducted in the absence of any commercial or financial relationships that could be construed as a potential conflict of interest.

## Publisher’s Note

All claims expressed in this article are solely those of the authors and do not necessarily represent those of their affiliated organizations, or those of the publisher, the editors and the reviewers. Any product that may be evaluated in this article, or claim that may be made by its manufacturer, is not guaranteed or endorsed by the publisher.

## References

[1] Schmidt E Zillikens D. Pemphigoid diseases. *Lancet.* (2013) 381:320–32. 10.1016/S0140-6736(12)61140-4 23237497

[2] Beek N Zillikens D Schmidt E. Bullous autoimmune dermatoses–clinical features, diagnostic evaluation, and treatment options. *Dtsch Arztebl Int.* (2021) 118:413–20. 10.3238/arztebl.m2021.0136 34369370 PMC8380840

[3] Kridin K Shihade W Bergman R. Mortality in patients with bullous pemphigoid: a retrospective cohort study, systematic review and meta-analysis. *Acta Derm Venereol.* (2019) 99:72–7. 10.2340/00015555-2930 29963683

[4] Habtemariam S. Berberine pharmacology and the gut microbiota: a hidden therapeutic link. *Pharmacol Res.* (2020) 155:104722. 10.1016/j.phrs.2020.104722 32105754

[5] Imenshahidi M Hosseinzadeh H. Berberine and barberry (*Berberis vulgaris*): a clinical review. *Phytother Res.* (2019) 33:504–23. 10.1002/ptr.6252 30637820

[6] Kuo CL Chi CW Liu TY. The anti-inflammatory potential of berberine *in vitro* and *in vivo*. *Cancer Lett.* (2004) 203:127–37. 10.1016/j.canlet.2003.09.002 14732220

[7] Imenshahidi M Hosseinzadeh H. Berberis vulgaris and berberine: an update review. *BMC Complement Altern Med.* (2016) 30:1745–64. 10.1002/ptr.5693 27528198

[8] Xiao CW Ji QA Wei Q Liu Y Bao GL. Antifungal activity of berberine hydrochloride and palmatine hydrochloride against microsporum canis –induced dermatitis in rabbits and underlying mechanism. *BMC Complement Altern Med.* (2015) 15:177. 10.1186/s12906-015-0680-x 26054937 PMC4460627

[9] Wojtyczka RD Dziedzic A Kępa M Kubina R Kabała-Dzik A Mularz T Berberine enhances the antibacterial activity of selected antibiotics against coagulase-negative staphylococcus strains *in vitro*. *Molecules.* (2014) 19:6583–96. 10.3390/molecules19056583 24858093 PMC6272005

[10] Zhang P He LB Zhang J Mei XF Zhang YY Tian H Preparation of novel berberine nano-colloids for improving wound healing of diabetic rats by acting Sirt1/NF-κb pathway. *Colloids Sur.* (2020) 187:110647. 10.1016/j.colsurfb.2019.110647 31761520

[11] Samadian H Zamiri S Ehterami A Farzamfar S Vaez A Khastar H Electrospun cellulose acetate/gelatin nanofibrous wound dressing containing berberine for diabetic foot ulcer healing: *in vitro* and *in vivo* studies. *Sci Rep.* (2020) 10:8312. 10.1038/s41598-020-65268-7 32433566 PMC7239895

[12] Bağcı IS Horváth ON Ruzicka T Sárdy M. Bullous pemphigoid. *Autoimmun Rev.* (2017) 16:445–55. 10.1016/j.autrev.2017.03.010 28286109

[13] Takegami Y Makino T Matsui K Ueda C Fukuda S Hashimoto T Coexistence of antilaminin-332-type mucous membrane pemphigoid, lamina lucida-type linear IGA bullous dermatosis and sjogren syndrome. *Clin Exp Dermatol.* (2013) 38:194–6. 10.1111/ced.12030 23397948

[14] Lucariello RJ Villablanca SE Mascaró JM Reichel M. Association between bullous pemphigoid and malignancy: a meta-analysis. *Australas J Dermatol.* (2018) 59:253–60. 10.1111/ajd.12764 29313891

[15] Liu SD Chen WT Chi CC. Association between medication use and bullous pemphigoid: a systematic review and meta-analysis. *JAMA Dermatol.* (2020) 156:891–900. 10.1001/jamadermatol.2020.1587 32584924 PMC7301306

[16] Chen XX Zhang YQ Luo ZC Wu YJ Niu TX Zheng JY Prognostic factors for mortality in bullous pemphigoid: a systematic review and meta-analysis. *PLoS One.* (2022) 17:e0264705. 10.1371/journal.pone.0264705 35427358 PMC9012347

[17] Terra JB Potze WJ Jonkman MF. Whole body application of a potent topical corticosteroid for bullous pemphigoid. *J Eur Acad Dermatol Venereol.* (2014) 28:712–8. 10.1111/jdv.12153 23551654

[18] Kalinska-Bienias A Kowalczyk E Jagielski P Kowalewski C Wozniak K. Tetracycline, nicotinamide, and lesionally administered clobetasol as a therapeutic option to prednisone in patients with bullous pemphigoid: a comparative, retrospective analysis of 106 patients with long-term follow-up. *Int J Dermatol.* (2019) 58:172–7. 10.1111/ijd.14270 30350359

[19] Kakuta R Yamagami J Funakoshi T Takahashi H Ohyama M Amagai M. Azathioprine monotherapy in autoimmune blistering diseases: a feasible option for mild to moderate cases. *J Dermatol.* (2018) 45:334–9. 10.1111/1346-8138.14173 29250862

[20] Sticherling M Franke A Aberer E Glaser R Hertl M Pfeiffer C An open, multicentre, randomized clinical study in patients with bullous pemphigoid comparing methylprednisolone and azathioprine with methylprednisolone and dapsone. *Br J Dermatol.* (2017) 177:1299–305. 10.1111/bjd.15649 28494097

[21] Williams HC Wojnarowska F Kirtschig G Mason J Godec TR Schmidt E Doxycycline versus prednisolone as an initial treatment strategy for bullous pemphigoid: a pragmatic, non-inferiority, randomised controlled trial. *Lancet.* (2017) 389:1630–8. 10.1016/S0140-6736(17)30560-3 28279484 PMC5400809

[22] Beissert S Werfel T Frieling U Böhm M Sticherling M Stadler R A comparison of oral methylprednisolone plus azathioprine or mycophenolate mofetil for the treatment of bullous pemphigoid. *Arch Dermatol.* (2007) 143:1536–42. 10.1001/archderm.143.12.1536 18087004

[23] Engineer L Ahmed AR. Role of intravenous immunoglobulin in the treatment of bullous pemphigoid: analysis of current data. *J Am Acad Dermatol.* (2001) 44:83–8. 10.1067/mjd.2000.112288 11148470

[24] Muller PA Brocker EB Klinker E Stoevesandt J Benoit S. Adjuvant treatment of recalcitrant bullous pemphigoid with immunoadsorption. *Dermatology.* (2012) 224:224–7. 10.1159/000339071 22678083

[25] Bishnoi A De D Handa S Mahajan R. Biologics in autoimmune bullous diseases: current scenario. *Indian J Dermatol Venereol Leprol.* (2021) 87:611–20. 10.25259/IJDVL_886_19 34245525

[26] Garrido P QueiróS C Travassos A Borges-Costa J Filipe P. Emerging treatments for bullous pemphigoid. *J Dermatolog Treat.* (2022) 33:649–61. 10.1080/09546634.2020.1782325 32536232

[27] Ge XL Li SZ Jin X Jin HZ Zuo YG. Treatment of bullous pemphigoid in Chinese patients with *Tripterygium wilfordii* Hook F. *Dermatol Ther.* (2020) 33:e13721. 10.1111/dth.13721 32500934

[28] Hu W Zhao J Li P Zhao GM. Clinical observation of postage stamp therapy against herpes zoster. *Chin J Clin Phys.* (2020) 48:495–7. 10.3969/j.issn.2095-8552.2020.04.038

